# Novel approach in treatment of *Burkholderia pseudomallei* osteomyelitis: salvage therapy with levonadifloxacin

**DOI:** 10.1093/jacamr/dlaf122

**Published:** 2025-07-09

**Authors:** Neha Gupta, Vikas Agashe, Rajeev Soman, Kandarp Vidyarthi, Rajiv Gupta, Ishita Sen, Mrinalini Koley, Balaji Veeraraghavan, Kailash Gupta

**Affiliations:** Department of Infectious Diseases, NG Swastha and Fortis Memorial Research Institute, Gurugram, India; Department of Orthopaedics, P. D. Hinduja and Agashe Hospital, Mumbai, India; Department of Infectious Diseases, Jupiter Hospital, Pune, India; Department of Orthopaedics, Paras Hospitals, Gurugram, India; Department of Radiology, Medanta, The Medicity, Gurugram, India; Department of Nuclear Medicine, Fortis Memorial Research Institute, Gurgaon, India; Department of Nuclear Medicine, Fortis Memorial Research Institute, Gurgaon, India; Department of Clinical Microbiology, Christian Medical College, Vellore, India; General Surgeon, Teerath Nursing Home, Rewa, Madhya Pradesh, India

## Abstract

**Background:**

Melioidosis, caused by *Burkholderia pseudomallei*, is a deadly infection with a case fatality rate of 10%–50%. Osteomyelitis, a rare manifestation, is particularly challenging due to diagnostic delays, misidentification, limited bone penetration of antibiotics and frequent relapses. Antibiotic intolerance emerges as a significant hurdle, with up to 26%–51% of patients developing severe allergies to trimethoprim/sulfamethoxazole, along with intolerability to ceftazidime and meropenem. These limitations demand innovative therapeutic approaches to combat this pathogen.

**Objectives:**

To report a novel case of *B. pseudomallei*-induced radial osteomyelitis successfully managed with levonadifloxacin, a broad-spectrum benzoquinolizine antibiotic, after intolerance to the conventional therapies.

**Methods:**

A 59-year-old man presented with radial osteomyelitis requiring multiple rounds of surgical debridement. Severe allergic reactions to trimethoprim/sulfamethoxazole and ceftazidime, and meropenem-associated leukopenia made traditional therapies unfeasible. Levonadifloxacin was introduced as a salvage therapy, leveraging its efficacy against *B. pseudomallei*, favourable bone penetration, antibiofilm activity and tolerability. The patient received IV levonadifloxacin for 5 days, followed by almost 8 months of its oral prodrug, alalevonadifloxacin.

**Conclusions:**

Levonadifloxacin emerged as the salvage therapy for this patient, achieving significant clinical improvement without relapse after more than 6 months of therapy cessation, despite the life-threatening combination of melioidosis-associated osteomyelitis and antibiotic intolerance. This first reported use of levonadifloxacin in melioidosis underscores its potential as an alternative therapy in such complex infections. Aggressive surgical intervention and tailored antimicrobial strategies remain crucial to overcoming these dual challenges.

## Introduction

Melioidosis, caused by *Burkholderia pseudomallei*, is a severe infectious disease with a case fatality rate of 10%–50%. Melioidosis can manifest in diverse clinical presentations, including community-acquired sepsis, pneumonia, soft tissue infections, deep suppurative abscesses and bone infections.^1,2^ While osteomyelitis is a rare manifestation, it carries significant morbidity and diagnostic challenges due to non-specific clinical presentations, delayed culture growth, and misidentification. Antibiotic intolerance, including adverse reactions to standard therapies like trimethoprim/sulfamethoxazole (seen in up to 26%–51% of patients), further complicates management.^3,4^ This report documents the first successful use of levonadifloxacin, a novel benzoquinolizine fluoroquinolone, as a salvage therapy in melioidosis-associated osteomyelitis. A part of this case was presented at 10^th^ World Melioidosis Congress in 2024.^5^

## Case presentation

A 59-year-old gentleman with no comorbidities presented with acute onset fever and gradually worsening right forearm pain. Initial investigations revealed elevated inflammatory markers [erythrocyte sedimentation rate (ESR): 35 mm/h; C-reactive protein (CRP): 61.5 mg/L]. He was empirically treated with cefixime elsewhere for suspected *Salmonella* infection, resulting in fever resolution but persistent pain.

**Figure 1.**
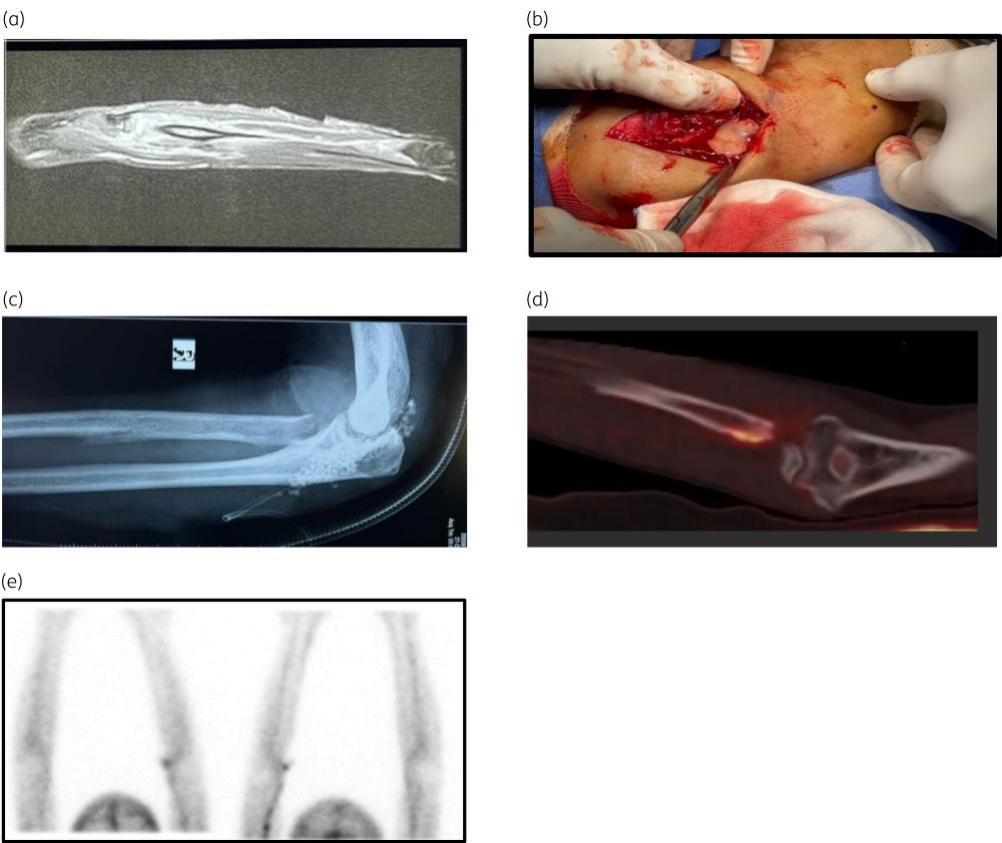
Images showing proximal radial osteomyelitis, surgical debridement, antibiotic stimulan placement and WBC scan. (a) Contrast MRI revealed proximal radial osteomyelitis with elbow joint effusion, (b) open debridement and radial head excision with frank pus coming out after the surgical incision, (c) antibiotic stimulan place, (d) ^18^F-FDG labelled autologous leucocyte PET CT scan, (e) bone scan with no uptake 3 weeks after discontinuation of the antibiotic.

Magnetic resonance imaging (MRI) of the forearm identified proximal radial osteomyelitis with joint effusion (Figure 1a). Despite these findings, an orthopaedic review recommended conservative management with antibiotics. However, symptoms worsened within 11 days, with recurrent fever and progressive forearm pain. Repeat imaging revealed extensive osteomyelitis with fracture of the radial head and severe soft tissue involvement (Figure S1, available as Supplementary data at *JAC-AMR* Online). A heterogeneously enhancing collection 7 × 22 × 23 mm around the neck of the radius extending into the supinator muscle and coracobrachialis was observed. Computed tomograpgy (CT) showed severe thinning of the head, neck and tuberosity of radius with heterogeneous attenuation and inflammation in the intermuscular and intramuscular soft tissues around the radius neck.

Repeat blood cultures grew Gram-negative bacilli after 37 h of incubation, which were initially identified as *Burkholderia* species using MALDI-TOF MS (Matrix-Assisted Laser Desorption/Ionization Time-of-Flight Mass Spectrometry; VITEK MS^®^, Knowledge Base version 3.2, bioMérieux, France), in accordance with the manufacturer’s instructions. Species-level identification was undertaken using a battery of biochemical tests (Figure S2 and Table S1), which was further supported by VITEK 2 Compact^®^ software version 9.02 (bioMérieux), confirming the presence of *B. pseudomallei*. Given that the patient was immunocompetent with no known comorbidities, environmental exposure was considered as the most likely source of *B. pseudomallei* infection. Recent construction activity near the patient’s residence had damaged the municipal pipeline water supply. This led to temporary reliance on domestic bore water, which had become visibly contaminated with drainage water. Manual chlorination of the contaminated domestic bore water was carried out, but it was likely not successful. While there is no definitive evidence, the likelihood of sub-chlorination in the household water supply was inferred based on sensory characteristics of the water; the typical pungent chlorine smell was noticeably milder than expected, and the water lacked the pale yellow or green hue commonly associated with adequately chlorinated water, suggesting inadequate disinfection. The onset of symptoms in November 2024 corresponds with the timeline of potential initial exposure, as the manual chlorination of the contaminated domestic bore water occurred in October 2024. This suggests an exposure-to-symptom onset period of approximately 3–4 weeks, as has been frequently reported. This seems to be the case of acquiring infection through ingestion of sub-chlorinated bore well water, known to be a well-established risk factor for *B. pseudomallei* exposure in endemic regions.^6–8^ However, microbiological testing of the domestic bore water used by the patient was not conducted.

Considering the antibiotic susceptibility test (AST) results (Table 1) and history of allergy to trimethoprim/sulfamethoxazole, initial treatment with IV meropenem and minocycline was initiated, coupled with surgical debridement (Figure 1b). Intraoperative cultures also grew *B. pseudomallei*. Despite therapy, persistent fever and worsening symptoms necessitated a second surgical debridement. Advanced surgical techniques, including intramedullary debridement and local ceftazidime-loaded calcium sulphate delivery (Figure 1c), were employed for optimal source control.

**Table 1.** Activity of levonadifloxacin and comparator antibiotics (AST) against *B. pseudomallei* recovered from blood sample of patient

Antibiotics	MIC (mg/L)
Levonadifloxacin	1 (S)^a^
Levofloxacin	4 (NA)
Meropenem	≤2 (NA)
Minocycline	≤2 (NA)
Ceftazidime	≤2 (S)
Trimethoprim/sulfamethoxazole	≤2/38 (S)
Amoxicillin/clavulanic acid	≤8/4 (S)

S, susceptible; NA, not available. The susceptibility of *B. pseudomallei* to levonadifloxacin and comparator antibiotics was determined using the broth microdilution method as recommended in CLSI M07-A12, 2024.

^a^PK/PD-based susceptibility breakpoint of ≤4 mg/L was employed. Interpretation as per CLSI criteria provided in CLSI M45, A2, 2010.^9^

Postoperatively, the patient was started on IV ceftazidime and oral trimethoprim/sulfamethoxazole but developed severe drug-related eosinophilia and systemic symptoms (DRESS) syndrome (Figure S3). Meropenem and minocycline were then initiated, but therapy was limited by minocycline-related anorexia and meropenem-induced leucopenia. These adverse reactions rendered standard therapies unfeasible.

Levonadifloxacin, a novel benzoquinolizine fluoroquinolone with proven activity against *B. pseudomallei*, was initiated as a salvage therapy. Supported by *in vitro* studies and robust pharmacokinetics, levonadifloxacin was administered IV for 5 days, followed by an oral regimen.^10–12^ This approach eliminated the need for a peripherally inserted central catheter line, significantly enhancing patient compliance.

The therapy was well tolerated, with no adverse effects, and led to marked clinical improvement. An ^18^F-fluorodeoxyglucose (FDG)-labelled autologous leucocyte positron emission tomography (PET) CT scan, done in August 2024, showed significant resolution, with only mild uptake at the medial proximal radius margin and no abnormal uptake elsewhere (Figure 1d). The antibiotic course was extended for an additional 2 months, achieving complete remission with no relapse. A repeat bone scan done in November 2024, after 3 weeks of discontinuation of antibiotics, revealed no evidence of infection (Figure 1e).

This represents the first documented case of long-term levonadifloxacin use in melioidosis, spanning 8.5 months (255 days), and underscores its potential as an alternative therapy in antibiotic-intolerant cases. As the relapse is often reported in such difficult-to-treat melioidosis infections, it is interesting to note that, more than 6 months post-therapy cessation, the patient remains clinically stable with no signs of recurrence. Blood tests, including biomarkers, are normal, reinforcing sustained remission.

## Ethics

Written informed consent was obtained from the patient, in accordance with COPE and ICMJE guidelines. Notably, the patient, who is also a surgeon, was actively involved in the manuscript’s preparation and has consented to its publication.

## Discussion

Melioidosis poses diagnostic and therapeutic challenges, particularly in the absence of classical symptoms. In this case, the initial misdiagnosis was attributed to atypical presentation and the absence of prior reports of *B. pseudomallei* in the patient’s region.^13,14^

In our case, the isolate was susceptible to trimethoprim/sulfamethoxazole, minocycline, ceftazidime, meropenem and amoxicillin/clavulanic acid. Given the patient’s initial presentation, empirical meropenem was started before *B. pseudomallei* identification. Following identification by MALDI-TOF MS, VITEK 2 Compact^®^ software version 9.02 and biochemical tests, and considering the patient’s past allergy to trimethoprim/sulfamethoxazole, minocycline was added as part of the tailored regimen.

After surgical intervention and definitive susceptibility testing, meropenem was de-escalated to ceftazidime. Trimethoprim/sulfamethoxazole was also introduced 5 days later, but the patient developed severe DRESS syndrome after a few weeks, necessitating discontinuation of both trimethoprim/sulfamethoxazole and ceftazidime. Attempts at reintroduction first with ceftazidime and later with trimethoprim/sulfamethoxazole were unsuccessful due to persistent intolerance. Subsequently, meropenem and minocycline were restarted, but the patient developed anorexia and leucopenia, limiting their long-term use. This posed a significant therapeutic challenge, necessitating the exploration of alternative treatment options to ensure both efficacy and tolerability. Management of melioidosis osteomyelitis requires prolonged antibiotic therapy and surgical intervention. Repeated debridement and local antibiotic delivery were critical in achieving source control. However, the patient’s intolerance to multiple antibiotics, including meropenem, trimethoprim/sulfamethoxazole and ceftazidime, presented a formidable challenge, highlighting the urgent need for alternative therapies.

Levonadifloxacin, a novel benzoquinolizine fluoroquinolone approved in India for treating acute bacterial skin and skin structure infections, diabetic foot infection and concurrent bacteraemia, offers superior pharmacokinetics, including high bone penetration, bactericidal activity, antibiofilm properties, and oral bioavailability and tolerability.^10–12,15,16^ It has been successfully used for treating difficult-to-treat bone and joint infections.^17^

A recent study highlights the potent *in vitro* activity of levonadifloxacin against *B. pseudomallei*, suggesting its potential as an alternative treatment for melioidosis.^10^ In this study, levonadifloxacin demonstrates activity superior to that of ciprofloxacin and various comparators. Time–kill assays reveal a rapid bactericidal action of levonadifloxacin, achieving an approximately 3 log_10_ bacterial kill, whereas doxycycline remains bacteriostatic. Recent pharmacokinetic studies in Wistar rats demonstrated levonadifloxacin’s rapid and effective distribution within bone tissues, with a bone-to-serum (B/S) ratio comparable across segments.^12^ Notably, after 5 days of repeated dosing, the B/S ratio increased to 1.14 in hard bone, which is about 5× higher than the unbound levonadifloxacin in plasma, indicating its excellent penetration crucial for treating bone infections. Moreover, levonadifloxacin attains significantly higher concentrations in epithelial lining fluid and alveolar macrophages, critical sites for *B. pseudomallei* persistence, particularly in acidic intracellular environments, where the bacteria can evade immune responses. Current oral therapies, such as trimethoprim/sulfamethoxazole and doxycycline, lack intracellular bactericidal efficacy.^4,18–20^

In our patient, levonadifloxacin served as a salvage therapy, overcoming antibiotic intolerance. With benchmark safety, successful application of levonadifloxacin in this case highlights its potential to revolutionize melioidosis management, particularly for patients with antibiotic resistance or intolerance, and opens new therapeutic avenues for such a challenging infection. Considering its robust pharmacokinetic and pharmacodynamic profile, levonadifloxacin coupled with remarkable safety, even in long-term administration, could be an appropriate candidate in both the acute and eradication phases of melioidosis.

We acknowledge that this is a single-patient case report, and while it highlights an innovative salvage therapy with levonadifloxacin available as both oral and IV dosages, broader case series or controlled studies are needed to strengthen the findings. As more cases and data become available, we aim to further evaluate its clinical utility.

### Conclusions

This case highlights the challenges of managing antibiotic-intolerant melioidosis osteomyelitis and the successful application of levonadifloxacin as a novel salvage therapy. In this patient, levonadifloxacin played a critical role in both the intensive and eradication phases without any safety concerns, ensuring sustained therapy and successful clinical resolution. Further studies are warranted to validate its efficacy in larger cohorts.

## Supplementary Material

dlaf122_Supplementary_Data

## Supplementary data

Figures S1 to S3 and Table S1 are available as Supplementary data at *JAC-AMR* Online.
