# Supplementary material for: Novel approach in treatment of Burkholderia pseudomallei osteomyelitis: salvage therapy with levonadifloxacin
Source: JAC Antimicrob Resist. 2025 Jul 9;7(4):dlaf122. doi: 10.1093/jacamr/dlaf122 (PMC12238538; doi:10.1093/jacamr/dlaf122)
Supplement: dlaf122_Supplementary_Data [file dlaf122_supplementary_data.docx]

**Title:**

Novel Approach in Treatment of Burkholderia pseudomallei Osteomyelitis: Salvage Therapy with Levonadifloxacin

**Authors and affiliations**

Neha GUPTA^1*^, Vikas AGASHE^2^, Rajeev SOMAN^3^, Kandarp VIDYARTHI^4^, Rajiv GUPTA^5^, Ishita SEN^6^, Mrinalini KOLEY^6^, Balaji VEERARAGHAVAN^7^, Kailash GUPTA^8^

^1^Department of Infectious Diseases, NG Swastha and Fortis Memorial Research Institute, Gurugram, India; ^2^Department of Orthopaedics, P. D. Hinduja and Agashe Hospital, Mumbai, India; ^3^Department of Infectious Diseases, Jupiter Hospital, Pune, India; ^4^Department of Orthopaedics, Paras Hospitals, Gurugram, India; ^5^Department of Radiology, Medanta, The Medicity, Gurugram, India; ^6^Department of Nuclear Medicine, Fortis Memorial Research Institute, Gurgaon, India; ^7^Department of Clinical Microbiology, Christian Medical College, Vellore, India; ^8^General Surgeon, Teerath Nursing Home, Rewa, Madhya Pradesh, India

***Corresponding author:**

**Dr. Neha Gupta**

Sr. Infectious Diseases Physician,

NG Swastha and Fortis Memorial Research Institute,

Gurugram, India.

Email: ngswastha@gmail.com

Short running title: Levonadifloxacin as a salvage therapy for meliodosis

**Figure S1.** Contrast MRI suggestive of progression of osteomyelitis

a) Progression of osteomyelitis increase in collection with extensive soft tissue edema; b) Distal radius involvement; c) Impending fracture of radius head

**Figure S2.** Blood culture grew colonies of *Burkholderia pseudomallei*

**
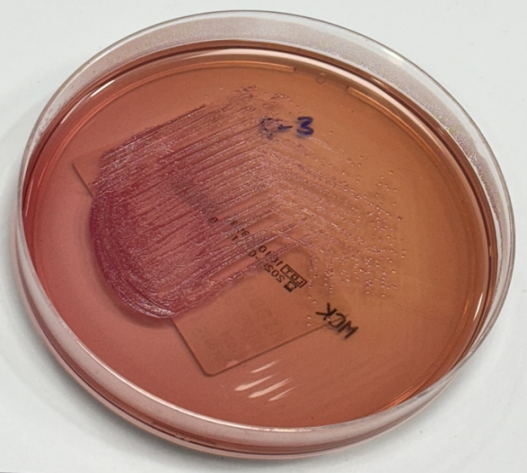
**

**Table S1.** Biochemical tests used for *B. pseudomallei* identification

| **Characters** | ***B. pseudomallei*** | ***B. cepacia*** |
| --- | --- | --- |
| Motility | + | + |
| Growth on MacConkey agar | + | + |
| Oxidase test | + | + |
| Oxidase-fermentation Glucose (oxidative) | + | + |
| Growth at 42˚C | + | + |
| Nitrate reduction with nitrogen gas | + | ― |
| Lysine Decarboxylate | ― | + |
| Arginine Dihydrolase | + | ― |
| Polymyxin (300 IU) susceptibility | Resistant | Resistant |
| Oxidase-fermentation Salicin (oxidative) | ― | + |
| Oxidase-fermentation Adonitol (oxidative) | ― | + |
| Reference: Koneman's color atlas and textbook of diagnostic microbiolgy by Gary W. Procop et al., 7^th^ Ed 2017, Pg no. 347 | | |

**Figure S3.** Severe pruritic rashes and DRESS with ceftazidime and trimethoprim/sulfamethoxazole

**
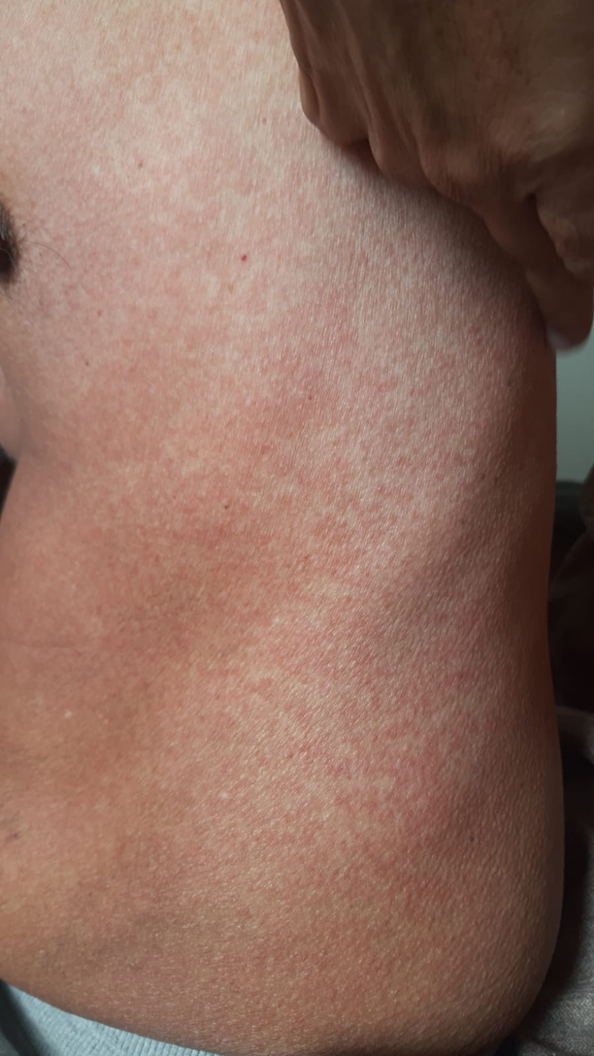
**
